# Accuracy of Mothers’ Perception of Birth Size to Predict Birth Weight Data in Bangladesh

**DOI:** 10.1007/s10995-024-03975-7

**Published:** 2024-08-23

**Authors:** Jahidur Rahman Khan, K. Shuvo Bakar, Nabil Awan, Olav Muurlink, Nusrat Homaira

**Affiliations:** 1 Discipline of Paediatrics, School of Clinical Medicine, University of New South Wales, Sydney, NSW 2031 Australia; 2 School of Public Health, Faculty of Medicine and Health, University of Sydney, Sydney, NSW 2006 Australia; 3 Department of Statistics, University of Wisconsin–Madison, Madison, WI USA; 4 School of Business and Law, Central Queensland University, Brisbane, Australia; 5 James P. Grant School of Public Health, BRAC University, Dhaka, Bangladesh

**Keywords:** Birth size, Birth weight, Agreement, Bangladesh

## Abstract

**Objectives:**

The prevalence of low birth weight (LBW) is an important indicator of child health and wellbeing. However, in many countries, decisions regarding care and treatment are often based on mothers’ perceptions of their children’s birth size due to a lack of objective birth weight data. Additionally, birth weight data that is self-reported or recorded often encounters the issue of heaping. This study assesses the concordance between the perceived birth size and the reported or recorded birth weight. We also investigate how the presence of heaped birth weight data affects this concordance, as well as the relationship between concordance and various sociodemographic factors.

**Methods:**

We examined 4,641 birth records reported in the 2019 Bangladesh Multiple Indicator Cluster Survey. The sensitivity-specificity analysis was performed to assess perceived birth size’s ability to predict LBW, while Cohen’s *Kappa* statistic assessed reliability. We used the kernel smoothing technique to correct heaping of birth weight data, as well as a multivariable multinomial logistic model to assess factors associated with concordance.

**Results:**

Maternally-perceived birth size exhibited a low sensitivity (63.5%) and positive predictive value (52.6%) for predicting LBW, but a high specificity (90.1%) and negative predictive value (93.4%). There was 86.1% agreement between birth size and birth weight-based classifications (*Kappa* = 0.49, indicating moderate agreement). Smoothed birth weight data did not improve agreement (83.4%, *Kappa* = 0.45). Of the sociodemographic factors, early marriage was positively associated with discordance (i.e., overestimation).

**Conclusions:**

An important consideration when calculating the LBW prevalence is that maternally perceived birth size is not an optimal proxy for birth weight. Focus should be placed on encouraging institutional births and educating community health workers and young mothers about the significance of measuring and recording birth weight.

**Supplementary Information:**

The online version contains supplementary material available at 10.1007/s10995-024-03975-7.

## Significance

Maternally-perceived birth size has been used to determine the LBW prevalence in many countries, including Bangladesh, where many or most births occur at home and birth weight data are not routinely collected. Research in Bangladesh has not investigated the value of perceived birth size in predicting birth weight, and earlier research in different countries has yielded inconsistent results. This study demonstrates that maternal perception of birth size has limited LBW prediction accuracy in Bangladesh. Consequently, it is imperative to exercise caution when estimating the prevalence of LBW based on data on perceived birth size, as LBW is a critical indicator of health outcomes and informs policy and interventions at both individual and national levels.

**Supplementary Information:**

The online version contains supplementary material available at 10.1007/s10995-024-03975-7.

## Introduction

Birth weight is a newborn’s initial weight after delivery. Low birth weight (LBW) infants, typically those under 2500 g, have a higher risk of negative health outcomes later in life in physiological, psychological, and neuropsychological domains (Bohnert & Breslau, 2008; Class et al., 2014; Taylor et al., 2004). Infections, developmental delays, and hypertension are also more prevalent among LBW infants (Belachew & Tewabe, 2020; Ediriweera et al., 2017; Hack et al., 2005; Hilaire et al., 2021). Thus, the prevalence of LBW may play an important role in determining policy as well as carer behaviour related to maternal and child health (e.g., adequate prenatal care, maternal nutrition), resource allocation (e.g., identify high risk populations to provide quality health care), and targeted intervention (e.g., enhance access to prenatal care and improving nutritional education).

Despite the importance of birth weight data, there is a scarcity of comprehensive data in many countries where a substantial proportion of children are born in locations with little institutional or formal professional support; there is a lack of infrastructure to collect the data. For example, the Bangladesh Multiple Indicator Cluster Survey (MICS) 2019 reports that roughly 46.4% of women aged 15–49 who gave birth in the last two years did so at home (Bangladesh Bureau of Statistics (BBS) and UNICEF Bangladesh, 2019), usually with a traditional birth attendant present. In such home births there is no systematic approach to measuring the birth weight, hence LBW is primarily measured by obtaining information from mothers’ subjective estimates of their infants’ birth size during household surveys. In the absence of clinical birth weight data, this subjective measure of birth size remains a proxy indicator (Khan et al., 2018), though it may be imprecise. Additionally, maternal perceptions of birth size vary by sociodemographic and cultural norms (Channon, 2011). However, some information on birth weight is available from various health surveys for children who are primarily delivered at any health facility (Blanc & Wardlaw, 2005), which may only provide an approximation of the prevalence of LBW among these births, not a national estimate. Blanc and Wardlaw’s careful analysis of different countries’ data which offer a mix of weighed and estimated births, suggests a need to adjust estimated birth weights in a systematic manner (Blanc & Wardlaw, 2005). Women who gave birth in health facilities are more likely to live in urban areas and have a high socioeconomic status, thus extrapolating population data from this sample may be deceptive. Furthermore, birth weight is not always recorded systematically, even for births that occur in health facilities; thus, these survey data rely on a combination of recorded and maternal recall data, which may, as Blanc and Wardlaw note, suffer from heaping, or digit preference (Blanc & Wardlaw, 2005).

Though birth size data are used to estimate LBW, little is known about how closely these perceptions of birth size correspond to birth weights recorded or recalled in different countries (Acharya et al., 2023; Islam, 2014; Mbuagbaw & Gofin, 2010; Nigatu et al., 2019). Studies from Ethiopia, Oman, Nepal, and Cameroon suggest that mothers’ assessment of infants’ birth size was an inaccurate and poor proxy for LBW (Acharya et al., 2023; Islam, 2014; Mbuagbaw & Gofin, 2010; Nigatu et al., 2019). However, a study conducted in Cambodia, Kazakhstan, and Malawi reported that, the mothers’ assessment of birth size is an adequate proxy for birth weight, even though it is essential to control societal influences on perception (Channon, 2011). Given the variability in these two measures across different countries, it is worthwhile to investigate their agreement in countries, like Bangladesh, where evidence is scarce, most children are born at home, and weighing scales are uncommon, even in the family home. Earlier research in Bangladesh utilised perceived birth size to estimate LBW prevalence and its associated factors in a nationwide sample (Khan et al., 2020), while another study employed birth weight in a subsample (Khan et al., 2018), but these studies did not investigate concordance between these two measures. Thus, this study aimed to determine the concordance between the perceived size of infants at birth and birth weight-based measures of LBW using a subsample of the national sample. The study also provides an insight into how heaping might impact records, by assessing the influence of heaping on concordance, as well as the relationship between concordance and sociodemographic factors in Bangladesh.

## Methods

### Study Setting and Design

This study used data from the 2019 Bangladesh Multiple Indicator Cluster Survey (MICS), a national survey that employed multi-stage stratified cluster sampling method to draw samples (Bangladesh Bureau of Statistics (BBS) and UNICEF Bangladesh, 2019). The survey collected birth weight data for births that occurred two years prior to the survey. Out of 9,285 recorded births, only 4,641 were analysed due to missing data in birth size (*n* = 76), birth weight (*n* = 4,491), birth weight data source (*n* = 74), TV watching (*n* = 2), and inadequate sample size in a survey cluster (*n* = 1). The analytic sample is not necessarily representative of all births during the study period because weighed and unweighed newborns have different sociodemographic backgrounds. Following the consideration of survey features (i.e., cluster, strata, and sampling weight), the sample size of 4,641 was changed to 4,673.

### Outcome Variables

#### Mother’s Perceived Size at Birth

Mothers of children were asked to classify their infants’ birth sizes as ‘very large’, ‘larger than average’, ‘average’, ‘smaller than average’ or ‘very small’, to assess mothers’ perceptions of infant size at birth (Bangladesh Bureau of Statistics (BBS) and UNICEF Bangladesh, 2019). To assess its predictive accuracy for LBW, this study categorised birth size into two categories: average or above average (‘very large’, ‘larger than average’ and ‘average’) and small (‘smaller than average’ and ‘very small’) (Nigatu et al., 2019).

#### Birth weight

This survey also collected birth weight data from written records (i.e., health cards) or the mother’s recall for births that occurred within two years of the survey interview. The birth weight was then classified as ‘LBW’ (if < 2.5 kg) and ‘non-LBW’ (if ≥ 2.5 kg) according to the World Health Organisation cut-off.

Then, birth weight data were normalised and classified into categories based on standard deviation (SD) to determine the agreement between the birth size and birth weight (Nigatu et al., 2019). An agreement variable with three response categories was created by matching perceived birth size and categorised normalised birth weight variables: ‘concordant’ (perceived infant size equal to the normalised birth weight category), ‘underestimate’ (perceived infant size smaller than the normalised birth weight category), and ‘overestimate’ (perceived infant size greater than the normalised birth weight category) (Nigatu et al., 2019).

### Study Variables

A set of additional variables was included based on existing literature and availability (Acharya et al., 2023; Islam, 2014; Mbuagbaw & Gofin, 2010; Nigatu et al., 2019). These variables included the mother’s age (years), early marriage (‘yes’ if a mother married before 18, ‘no’ otherwise), educational status (pre-primary or none, primary, secondary, higher secondary or upper), media exposure (‘yes’ if she had at least one form of mass media exposure, such as watching TV, listening to the radio, or reading newspapers/magazines otherwise ‘no’), child’s sex (male, female), place of birth (home, health facility), source of birth weight (card, recall), household wealth status based on wealth index quintile (poorest, poorer, middle, richer, richest), and place of residence (rural, urban). The household wealth index is a composite metric calculated using principal component analysis on household features and assets (Bangladesh Bureau of Statistics (BBS) and UNICEF Bangladesh, 2019).

### Statistical Analysis

Analytic sample distributions were described using descriptive statistics. We calculated the average birth weight for each perceived birth size class and used the Kruskal-Wallis’s test to assess the statistical significance of differences in average birth weight across these classes. A sensitivity-specificity analysis was performed to determine the accuracy of the mother’s perceived infant birth size as an indicator of LBW, and the following metrics were calculated: sensitivity, specificity, positive predictive value (PPV), and negative predictive value (NPV) (Nigatu et al., 2019). Cohen’s *Kappa* was used to estimate the level of agreement between birth size- and weight-based measures of LBW, while Landis and Koch’s benchmark was used to evaluate relative strength (≤ 0 = poor, 0.01–0.20 = slight, 0.21–0.40 = fair, 0.41–0.60 = moderate, 0.61–0.80 = substantial, and 0.81–1.00 = almost perfect) (Landis & Koch, 1977). Then, a multivariable multinomial logistic regression model examined the relationship between sociodemographic factors and the agreement variable, selecting factors based on their bivariate relationship at 10% significance in an adjusted Wald test. Model results were presented as an adjusted odds ratio (AOR) with a 95% confidence interval (CI).

Density graphs were used to depict the birth weight distribution and data heaping to the terminal digits. Then, we constructed a digit preference variable (‘yes’ if the birth weight had a terminal digit of 0/5 and ‘no’ otherwise) and examined its association with data sources. The birth weight data were smoothed using a kernel density smoothing approach, and the aforementioned analyses were redone to see whether the findings changed. Data management and analysis were done in R 4.1.0.

### Ethical Approval

This study is based on publicly available Bangladesh MICS 2019 datasets. Permission to access and utilise these datasets were obtained from the UNICEF/MICS website (http://mics.unicef.org/surveys), so no additional ethical approval was required.

## Results

Most of the mothers were aged 20–29 years and married before 18 years (Table 1). The average birth weight was 3 kg (SD ± 0.6 kg), with 14.8% of infants weighing less than 2.5 kg and 17.9% being small or very small according to mothers’ perceived birth size. Mothers’ recall accounted for 88.8% of birth weight data, while health cards provided 11.2%.

**Table 1:** Distribution of analytic sample

Variables	Categories	%
Mother’s age (years)	15–19	14.1
	20–29	61.8
	30–39	22.9
	40–49	1.2
Early marriage	Yes	55.1
	No	44.9
Place of residence	Urban	27.9
	Rural	72.1
Household wealth status	Poorest	10.2
	Second	14.7
	Middle	19.1
	Fourth	23.8
	Richest	32.1
Mother’s education	Pre-primary or none	3.6
	Primary	14.4
	Secondary	54.4
	Higher secondary or upper	27.6
Mother’s media exposure	No	23.2
	Yes	76.8
Sex of child	Male	54.0
	Female	46.0
Place of delivery	Health facility	93.5
	Home	6.5
Birth weight	< 2.5 kg	14.8
	≥ 2.5 kg	85.2
Source of birth size data	From card	11.2
	From recall	88.8
Perceived birth size	Very large	1.8
	Larger than average	13.1
	Average	67.2
	Smaller than average	15.6
	Very small	2.3

### Accuracy of Mothers’ Perceived Birth Size to Predict LBW

Average birth weights varied significantly (p-value < 0.001) across mothers’ perceived birth size categories, with the average birth weight dropping from 3.84 kg to 1.79 kg when comparing very large to very small birth sizes (Table 2).

**Table 2:** Mean observed birth weight by mother’s perceived infant size at birth

	Observed birth weight		
Perceived birth size	Mean	SE	p-value
Very large	3.84	0.0747	< 0.001
Larger than average	3.57	0.0248	
Average	3.00	0.0097	
Smaller than average	2.38	0.0248	
Very small	1.79	0.0529	

SE: standard error

Table 3 compares birth size categories (smaller, average or above) with birth weight categories (LBW: < 2.5 kg, non-LBW: ≥ 2.5 kg). About 63.5% of LBW infants (birth weight < 2.5 kg) were also perceived as smaller than average or very smaller at birth (sensitivity = 63.5%). About 90.1% of non-LBW infants (birth weight ≥ 2.5 kg) were also perceived average or larger size at birth by their mothers (specificity = 90.1%). About 52.6% of infants perceived as smaller than average or very small by their mothers had birth weight < 2.5 kg (PPV = 52.6%), while about 93.4% of infants perceived as average or above-average birth size had birth weight ≥ 2.5 kg (NPV = 93.4%). The proportion of agreement (i.e., concordance) between perceived birth size (small and average or above) and birth weight (LBW and non-LBW) was 86.1%, with a *Kappa* value of 0.49 (moderate agreement). Importantly, non-LBW infants contributed more to this concordance value than LBW infants. There were about 14% discordant cases (8.5% overestimates and 5.4% underestimates). The *Kappa* statistic values for “card” and “recall” data were 0.55 and 0.48, respectively, indicating moderate agreement and no notable changes in agreement class based on birth weight data source (results not shown).

**Table 3:** Accuracy of mothers’ perceived infant birth size to predict low birth weight

	Observed birth weight		
	< 2.5 kg	≥ 2.5 kg	Total (%)
**Perceived birth size**			
Small	439 (9.4%)	396 (8.5%)	835 (17.9%)
Average or above	252 (5.4%)	3,586 (76.7%)	3,838 (82.1%)
**Total (%)**	691 (14.8%)	3,982 (85.2%)	4,673
**Indicator accuracy**			
Sensitivity	63.5		
Positive predictive value (PPV)	52.6		
Specificity	90.1		
Negative predictive value (NPV)	93.4		
**Agreement**			
Concordant	86.1		
Overestimate	8.5		
Underestimate	5.4		
Kappa (95% CI)	0.49 (0.45–0.53)		

When perceived newborn size (measured by five categories) was compared to normalised birth weights, 69% of the cases were concordant, 16% were underestimates, and 14.9% were overestimates (Table 4). The *Kappa* = 0.33 means there was a moderate level of agreement between the two metrics.

**Table 4:** Accuracy of mothers’ perceived infant birth size to predict low birth weight (five class)

	Birth weight (five classes)					
Birth size (five classes)	Very small	Smaller than average	Average	Larger than average	Very large	Total
Very small	55	33	19	1	0	107
Smaller than average	68	253	392	10	4	727
Average	8	186	2,723	186	38	3,142
Larger than average	1	5	367	172	66	612
Very large	0	0	40	22	22	84
Total	132	478	3,541	392	130	4,673
**Agreement**						
Concordant			69			
Overestimate			14.9			
Underestimate			16			
Kappa (95% CI)			0.33 (0.30–0.36)			

### Factors Associated with Mothers’ Perceived Birth Size and Weight Agreement

The regression model showed that early marriage was the only significant predictor of birth size-weight discordance (Table 5). Mothers who married before 18 (i.e. early marriage) were more likely to overestimate their children’ birth size (AOR 1.25, 95% CI [1.03, 1.51]).

**Table 5:** Associations between different factors and mothers’ reported infant birth size and birth weight agreement

Variables	Birth size and birth weight agreement (Base: Concordance)	
	Overestimate	Underestimate
	AOR (95% CI)	AOR (95% CI)
**Early marriage**		
No	1.00	1.00
Yes	1.25 (1.03, 1.51)	0.96 (0.79, 1.17)
**Mother’s education**		
Pre-primary or none	1.00	1.00
Primary	1.20 (0.69, 2.08)	0.80 (0.49, 1.30)
Secondary	1.05 (0.61, 1.78)	0.77 (0.49, 1.20)
Higher secondary or upper	0.86 (0.49, 1.50)	0.81 (0.50, 1.30)
**Household wealth status**		
Poorest	1.00	1.00
Second	0.97 (0.69, 1.36)	1.02 (0.72, 1.45)
Middle	0.81 (0.57, 1.14)	0.89 (0.63, 1.28)
Fourth	0.90 (0.64, 1.20)	0.99 (0.69, 1.42)
Richest	0.75 (0.52, 1.08)	0.93 (0.65, 1.33)
**Place of delivery**		
Health facility	1.00	1.00
Home	1.26 (0.91, 1.74)	0.84 (0.58, 1.22)
**Mother’s media exposure**		
No	1.00	1.00
Yes	0.93 (0.74, 1.17)	0.96 (0.76, 1.21)

AOR: adjusted odds ratio; CI: confidence interval

### Assessment of Birth weight Data for Potential Measurement Error

**Fig. 1:**
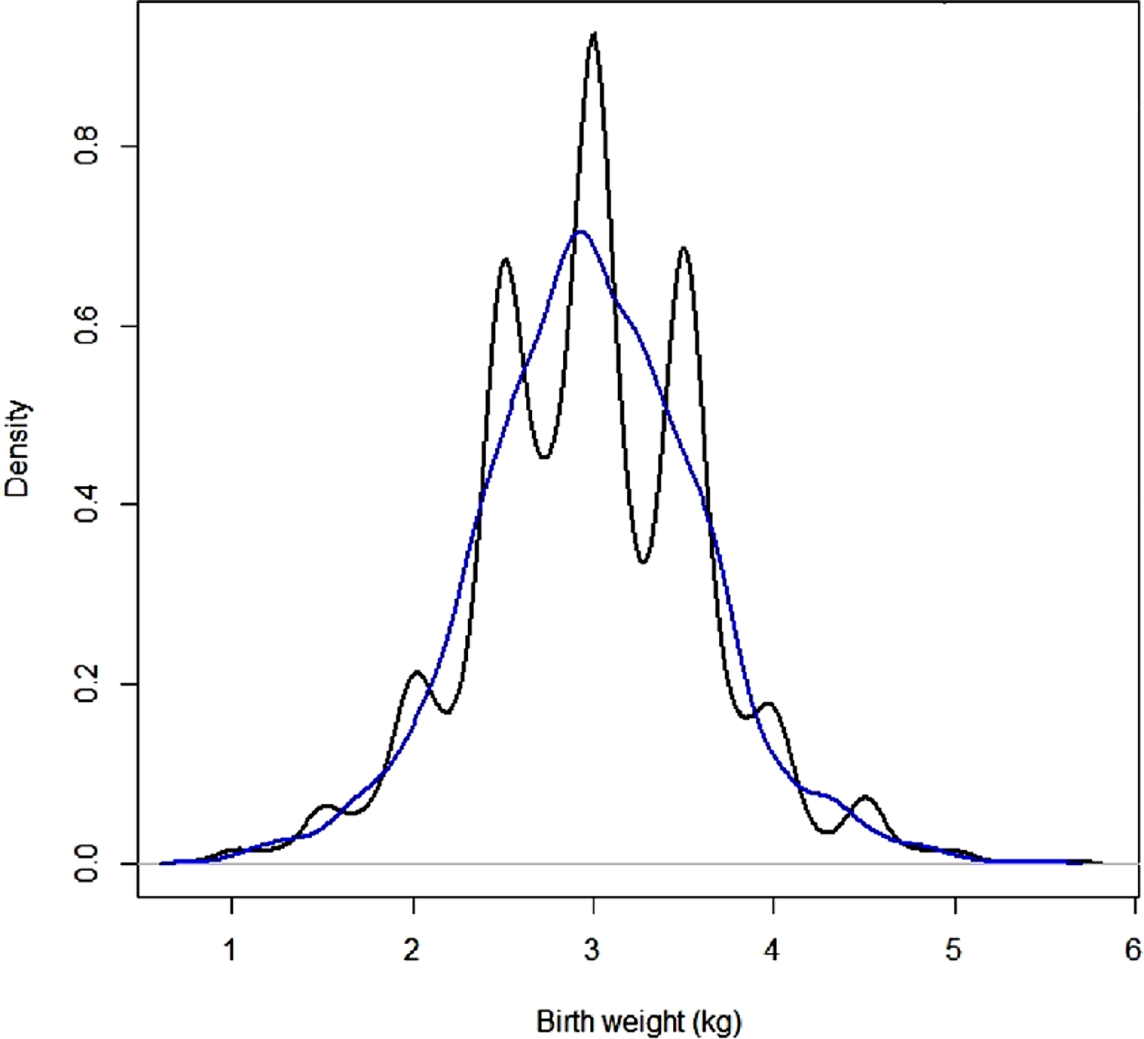
Density plot of observed birth weight (black) and smoothed birth weight (blue) data

Birth weight data was heaped to the terminal digits ‘0’ and ‘5’ (Fig. 1), with recall data exhibiting more heaping than health card data (Figure S1). However, the birth weight data source did not demonstrate a significant association with digit preference (results not shown). Kernel smoothing for heaping problem changed the distribution of birth weight data, as seen in Fig. 1. Smoothed data showed 19.1% LBW newborns (Table S1).

### Accuracy of Mothers’ Perceived Birth Size to Predict LBW Based on Smoothed Birth weight Data

The average smoothed birth weight decreased from 3.79 kg to 1.85 kg as birth size moved from very large to very small (Table S2). Table S3 compares the mother’s perceived birth size categories (smaller, average, or above) with smoothed birth weight categories (LBW: < 2.5 kg, non-LBW: ≥ 2.5 kg). The values of prediction statistics changed as birth weight data were smoothed. The sensitivity decreased by approximately 10%, while the specificity remained nearly unchanged. The NPV decreased by 4.2% while the PPV increased by 4.4%. The perceived birth size-smoothed birth weight agreement dropped to 83.4% (*Kappa* = 0.45, moderate agreement). Discordant cases were 16.6% (7.7% overestimates and 8.9% underestimates). When the five categories of birth size were compared to normalised smoothed birth weights, there were 66.3% cases of concordance (*Kappa* = 0.30, fair agreement), 17.4% cases of underestimation, and 16.3% cases of overestimation (Table S4). Heaping increased the fraction of discordant estimates, with underestimations increasing higher.

### Factors Associated with Mothers’ Perceived Birth Size and Smoothed Weight Agreement

Early married mothers were more likely than their counterparts to overestimate their infants’ birth size (AOR 1.24, 95% CI [1.03, 1.49]) (Table S5). The odds of overestimated birth size was higher among female infants than male infants (AOR 1.20, 95% CI [1.01, 1.43]). Mothers with at least higher education (AOR 0.61, 95% CI [0.38, 0.99]) and exposure to media (AOR 0.81, 95% CI [0.66, 1.00]) had lower odds of overestimation.

## Discussion

This study found that only 63.5% of perceived small-sized infants were LBW, dropping to 53.4% in smoothed birth weight data suggesting that using birth size as a measure of LBW may misclassify a significant portion of LBW infants. Furthermore, sociodemographic factors (e.g., early marriage) play a role in discordance while assessing LBW from maternal perceived birth size.

In Bangladesh, mothers’ perceived birth size had low sensitivity (63.5%) and PPV (52.6%) for predicting LBW infants. Mothers often failed to identify LBW infants as small size infants, and conversely. The sensitivity and PPV changed to 53.4% and 57.0%, respectively, following the smoothing of birth weight data heaping. Clearly, the classification remains unresolved. These findings (i.e., low sensitivity and PPV) are supported by earlier research in different countries (Acharya et al., 2023; Islam, 2014; Mbuagbaw & Gofin, 2010; Nigatu et al., 2019). Maternally-perceived birth size predicted non-LBW infants as average or large in a high proportion of cases, as evidenced by high specificity and NPV values. The high rates of identifying non-LBW infants resulted in high concordances between maternally-perceived birth size and birth weight-based LBW measures (i.e., 86.1% for observed data and 83.4% for heaping corrected data). However, the *Kappa* coefficients showed only moderate agreement (*Kappa* = 0.49 for observed data and *Kappa* = 0.45 for heaping corrected data) between perceived birth size- and weight-based LBW measures, which is consistent with findings from earlier research (Acharya et al., 2023; Islam, 2014; Mbuagbaw & Gofin, 2010; Nigatu et al., 2019). The agreement level decreased from moderate to fair (*Kappa* = 0.33 for observed data and *Kappa* = 0.30 for heaping corrected data) when analysing data based on five categories of birth size and weight. Thus, using maternally-perceived birth size as a proxy indicator to quantify the prevalence of LBW may misreport its magnitude and its contribution to child mortality, wellbeing, and future health and economic burden. Misreporting (e.g., underestimating) the LBW prevalence may contribute to the lack of prioritisation of nutrition and public health intervention for LBW newborns. Thus, caution should be exercised when using perceived birth size to measure LBW prevalence. Different factors may relate to the discordance between maternal birth size assessment and birth weight.

Mothers who were married before the age of 18 were more likely to classify their child as being larger than actual birth weight, possibly due to limited experience with motherhood. Their familial and community environments may have a greater impact, for example, children displaying significant body fat are frequently considered healthy and ‘large’ even though they are objectively small. Over 50% of our sample fall into this category—married before the age of 18— and this study is, if anything, likely to underestimate the prevalence of early marriage. Child marriage is illegal but common in Bangladesh, and when marriages are registered, particularly in rural communities, it is common for parties to be untruthful about the age of the bride (Nahid, 2014). A range of other factors (i.e., child sex, mothers’ education, and media exposure) were associated with birth size-smoothed birth weight discordance. Importantly, educated mothers had lower odds of overestimating the birth size of their children compared to non-educated or less educated mothers, which supports findings from an earlier study in Ethiopia (Nigatu et al., 2019). It is plausible that educated mothers may have a better understanding of the relationship between newborn birth size and birth weight, and a better ability to recall numbers (i.e., lesser recall bias). Media exposure had a similar impact.

Despite using national data there are a range of limitations to consider when interpreting the results of this study. The analytic data was a subsample because all infants’ birth weights were not known, so the findings should not be extrapolated to the entire population. Birth weight (recall and recorded) data were likely to be available only for wealthy and educated mothers and for births that occurred health facilities thus skewing the sample to some degree. Second, other dimensions of size (e.g., length and subcutaneous fat) may influence mother’s birth size perception; however, this survey data does not illuminate these additional dimensions. Finally, heaping data were corrected using a smoothing technique that was not compared to alternative approaches (e.g., mixture distribution, missing value imputation), so, the performance of all methods was unknown. Moreover, comparing various techniques was beyond the scope of this study and is unlikely to affect the overall results but may inspire future research.

This study holds significance for policymakers and researchers in countries like Bangladesh, where home births are prevalent, birth weight data is inadequate, and maternal perceived birth size is an unsatisfactory substitute indicator for birth weight. Several potential strategies can enhance the measurement of LBW. Promoting women’s utilisation of health facilities during childbirth can enhance the accuracy of birth weight measurement and decrease dependence on maternal recall or perceived birth size. This approach has the potential to enhance maternal-child care and reduce the likelihood of complications. It is imperative to ensure accessibility of health facilities to promote utilisation. Educating and training community health workers and traditional birth attendants on birth weight measurement and ensuring the availability of low-cost and simple measuring equipment can improve birth weight records. Community-based maternal and child health education programs can increase awareness regarding birth weight significance and encourage the use of skilled care during delivery. Public health campaigns leveraging the popular media may also be effective and adds to the body of work confirming the value of education in improving child health. The paper also suggests that triangulating birth weight data from multiple sources can be beneficial when the data is not available. Lastly, conducting validation studies to compare maternally perceived birth size with birth weight can aid in determining maternal perception accuracy.

## Conclusions

The study found a notable discordance between maternal perceived birth size and recorded birth weight as a measure of LBW. It suggests that maternal perception of birth size is an unreliable indicator of LBW. Early marriage has been found to be associated with discordance. The emphasis should be on promoting institutional births and providing education to community health workers and young mothers regarding the importance of measuring and recording birth weight accurately. Both formal education and media campaigns could play an important role in improving the accuracy of LBW estimation.

## Electronic Supplementary Material

Below is the link to the electronic supplementary material.

Supplementary Material 1

## Data Availability

The UNICEF/MICS website (http://mics.unicef.org/surveys) provides public access to this survey datasets.
