# Supplementary material for: Accuracy of Mothers’ Perception of Birth Size to Predict Birth Weight Data in Bangladesh
Source: Matern Child Health J. 2024 Aug 23;28(10):1677–84. doi: 10.1007/s10995-024-03975-7 (PMC11420260; doi:10.1007/s10995-024-03975-7)
Supplement: Supplementary file 1 — Supplementary Material 1 [file 10995_2024_3975_MOESM1_ESM.docx]

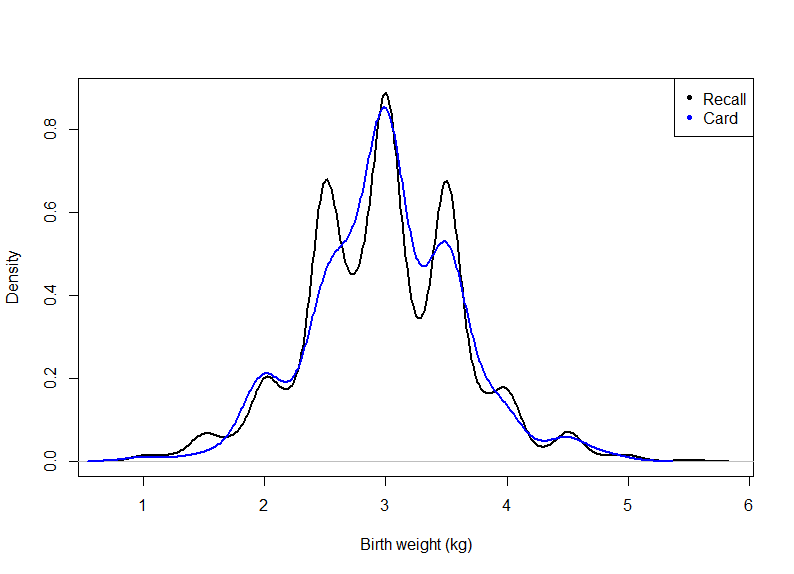


**Figure S1.** Density plot of birthweight by source of birthweight.

**Table S1.** Distribution of smoothed birthweight data.

|  | **Categories** | **%** |
| --- | --- | --- |
| Smoothed birthweight | <2.5 kg | 19.1 |
|  | ≥2.5 kg | 80.9 |

**Table S2.** Mean smoothed birthweight by mothers’ perceived infant size at birth.

|  | **Smoothed birth weight** |  |  |
| --- | --- | --- | --- |
| **Perceived baby size** | **Mean** | **SE** | **p-value** |
| Very large | 3.79 | 0.0774 | <0.001 |
| Larger than average | 3.54 | 0.0245 |  |
| Average | 3.00 | 0.0098 |  |
| Smaller than average | 2.40 | 0.0243 |  |
| Very small | 1.85 | 0.0529 |  |

SE: standard error

**Table S3.** Accuracy of mothers’ perceived infant birth size to predict low birthweight.

|  | **Smoothed birthweight** | |  |
| --- | --- | --- | --- |
|  | <2.5 kg | ≥2.5 kg | Total (%) |
| **Perceived birth size** |  |  |  |
| Small | 476 (10.2%) | 359 (7.7%) | 835 (17.9) |
| Normal or above | 416 (8.9%) | 3422 (73.2%) | 3838 (82.1) |
| **Total (%)** | 892 (19.1%) | 3781 (80.9%) | 4673 |
| **Indicator accuracy** |  | | |
| Sensitivity | 53.4 | | |
| PPV | 57.0 | | |
| Specificity | 90.5 | | |
| NPV | 89.2 | | |
| **Agreement** |  | | |
| Concordant | 83.4 | | |
| Overestimate | 7.7 | | |
| Underestimate | 8.9 | | |
| Kappa (95% CI) | 0.45 (0.41-0.49) | | |

**Table S4.** Accuracy of mothers’ perceived infant birth size to predict low birthweight using smoothed data (five class).

|  | **Smoothed birthweight (five classes)** | | | | | |
| --- | --- | --- | --- | --- | --- | --- |
| **Birth size (five classes)** | **Very small** | **Smaller than average** | **Average** | **Larger than average** | **Very large** | **Total** |
| Very small | 47 | 43 | 17 | 1 | 0 | 107 |
| Smaller than average | 75 | 251 | 384 | 14 | 4 | 727 |
| Average | 9 | 260 | 2591 | 244 | 38 | 3142 |
| Larger than average | 1 | 11 | 345 | 186 | 69 | 612 |
| Very large | 0 | 0 | 41 | 20 | 23 | 84 |
| Total | 131 | 565 | 3378 | 464 | 134 | 4673 |
| **Agreement** |  |  |  |  |  |  |
| Concordant |  |  | 66.3 | |  |  |
| Overestimate |  |  | 16.3 | |  |  |
| Underestimate |  |  | 17.4 | |  |  |
| Kappa (95% CI) |  |  | 0.30 (0.27-0.33) | |  |  |

**Table S5.** Associations between different factors and mothers’ reported infant size and smoothed birthweight agreement.

| **Variables** | **Birth size and birthweight (smoothed) agreement**  **(Base: Concordance)** | |
| --- | --- | --- |
|  | **Overestimate** | **Underestimate** |
|  | **AOR (95% CI)** | **AOR (95% CI)** |
| **Sex** |  |  |
| Male | 1.00 | 1.00 |
| Female | 1.20 (1.01, 1.43) | 0.95 (0.79, 1.14) |
| **Early marriage** |  |  |
| No | 1.00 | 1.00 |
| Yes | 1.24 (1.03, 1.49) | 1.05 (0.86, 1.27) |
| **Mother’s education** |  |  |
| Pre-primary or none | 1.00 | 1.00 |
| Primary | 0.92 (0.56, 1.52) | 1.06 (0.64, 1.76) |
| Secondary | 0.76 (0.48, 1.21) | 0.91 (0.57, 1.44) |
| Higher secondary or upper | 0.61 (0.38, 0.99) | 1.03 (0.64, 1.66) |
| **Place of delivery** |  |  |
| Health facility | 1.00 | 1.00 |
| Home | 1.22 (0.88, 1.68) | 0.81 (0.57, 1.17) |
| **Mother’s media exposure** |  |  |
| No | 1.00 | 1.00 |
| Yes | 0.81 (0.66, 1.00) | 0.86 (0.70, 1.05) |

AOR: adjusted odds ratio; CI: confidence interval.
